# Diagnosis and treatment for anal fistula: a systematic review of clinical practice guidelines and consensus statements

**DOI:** 10.3389/fsurg.2025.1566130

**Published:** 2025-07-02

**Authors:** Min-Yuan Lu, Jing Wang, Zhao-Chu Wang, Zhao-Lian Cai, Ning Liang, Rong Shi

**Affiliations:** ^1^ Department of Anorectal Surgery, The Affiliated People’s Hospital of Fujian University of Traditional Chinese Medicine, Fuzhou, China; ^2^ Department of Anorectal Surgery, Fuzhou Hospital of Traditional Chinese Medicine, Fuzhou, China; ^3^ Institute of Basic Research in Clinical Medicine, China Academy of Chinese Medical Sciences, Beijing, China

**Keywords:** diagnosis, guidelines, anal fistula, therapeutic, systematic review

## Abstract

**Background:**

Anal fistula constitutes a pathological channel originating either from the anal canal or rectum to the skin surrounding the anus, primarily characterized by recurrent pain, purulent discharge, and pruritus. This study aims to compare and standardize the recommendations for the diagnosis and treatment of anal fistula, drawing on contemporary clinical practice guidelines.

**Methods:**

A comprehensive search was conducted across multiple databases including PubMed, EMBASE, China National Knowledge Infrastructure, Wanfang Database, Chinese Science and Technology Periodical Database, and Chinese Biological Medicine Database, from their inception through April 1, 2024. The objective was to collate all published guidelines on anal fistula. The quality of the eligible guidelines was appraised by two reviewers using the Appraisal of Guidelines for Research and Evaluation II instrument.

**Results:**

The search yielded fifteen guidance documents —comprising nine guidelines and six consensus statements —each offering specific recommendations. Twelve of these documents address screening and diagnosis of anal fistula, while all fifteen discuss various treatment and management strategies. Document analysis highlighted MRI as the predominant diagnostic recommendation. Treatment and management strategies were categorized into four categories: preoperative management, surgical method selection, pharmacological interventions, and postoperative management. Regarding surgical interventions, all guidelines address incision and drainage of fistulas. Most guidelines offer a low recommendation for cutting setons, mainly attributed to the presence of incontinence. For high–positioned anal fistula, a push-pull flap procedure is recommended, whereas the LIFT procedure is advocated for newly identified, high, and sphincter-penetrating fistulas. Among the 15 guidelines and consensus statements evaluated in this study, more than half demonstrated methodological limitations, with particularly deficient performance in the applicability domain. As a critical determinant of implementation effectiveness, these deficiencies may undermine guidelines' capacity to optimize health outcomes.

**Conclusion:**

There is a pressing need for an updated search of potential evidence on the diagnosis and treatment of anal fistula. Effective diagnoses and therapeutic approaches, whether conventional or complementary and alternative medicine, should be thoroughly evaluated and incorporated based on robust evidence.

## Introduction

An anal fistula is a pathological channel connecting the anal or rectum to the skin surrounding the anus, primarily characterized by recurrent pain, purulent discharge, and pruritus. Occurring predominantly in males, its incidence peaks between 20 and 40 (1). Traditionally, surgical removal has been the definitive treatment for anal fistulas. Since Parks' 1976 elucidation of the anatomical relationship between anal fistulas and the anorectal muscles (2), therapeutic approaches have evolved significantly. The traditional fistulotomy for anal fistulas is now contested by sphincter-sparing techniques such as the Ligation of the Intersphincteric Fistula Tract (LIFT), rectal advancement flap, and the use of bioprosthetic fistula plugs, which aim to maintain sphincter integrity while effectively treating fistulas (3–5). Despite the absence of a universally accepted treatment standard, tailored approaches guided by high-quality clinical practice guidelines (CPGs) are crucial for optimal outcomes. According to research by Sackett DL (6), CPGs are formed by the best available evidence while incorporating healthcare provider experiences and the preferences of patients (7). Despite numerous publications of CPGs and expert consensus statements on anal fistulas, discrepancies due to varying evidence quality, regional differences, and the evolution of time undermine the guidelines' directive efficacy and pose challenges for the practitioners' decision-making processes (8, 9). It is thus imperative to systematically analyze and evaluate the quality evaluation of existing anal fistula guidelines. This endeavor aims to accurately and objectively analyze guideline recommendations, highlighting both congruences and divergences. Our objective is to provide a comprehensive mapping of current CPGs, thereby streamlining recommendations for the diagnosis and treatment of anal fistula, aiding clinicians in informed decision-making and assisting guideline developers in identifying areas for future updates.

## Materials and methods

This investigation involved a systematic review of published guidelines pertinent to anal fistula, as per the Statement 2020 for systematic evaluation reports of clinical practice guidelines (10). The quality and content of these guidelines were thoroughly assessed using the AGREE II instrument (11).

## Literature search

The databases searched included PubMed, EMBASE, Web of Science, and Medline. Additional searches were conducted by researchers ZC Wang and MY Lu through reputable sources including UK National Institute for Health and Clinical Excellence, the International Guidelines Collaboration Network, WHO, and the International Platform for Practice Guideline Registries for anal fistula guidelines. The search spanned from April 1, 2014, to April 1, 2024, mirroring methodologies utilized in studies by W.Y. Haw (12) and Bernd W.M. Arents (8). Search criteria were unbounded by language, focusing on titles, abstracts, and keywords with terms related to anal fistulas, guidelines, and consensus.

## Eligibility criteria

Included were guidelines on anal fistula issued by local, national, international or coalition governmental organizations. Excluded were systematic evaluations, reviews and other non-guideline literature, with duplicate searches removed. Guidelines issued in different periods were included only in their latest versions.

## Literature selection and data extraction

Independent guideline searches were executed by two researchers ZC Wang and MY Lu, who then screened the guidelines, resolving discrepancies in collaboration with a third researcher, R Shi. Extracted data encompassed guideline development details (development group, country, year, and guideline versions), scope and content (target population, diagnosis, and treatment), evidence support (systematic search and number of references), grading systems of recommendations (evidence level, recommendation strength), and conflict of interest (type of funding).

The included guidelines were reviewed, and the recommendations were extracted. Information on the level of evidence and strength of recommendations for diagnosis (or screening) and treatment (or management) were extracted separately. In cases where multiple versions of a guideline existed, but were authored by the same organization or group, recommendations specifically concerning the diagnosis and treatment of anal fistula were extracted from the latest version.

## Guidelines evaluation methodology

Quality of the included guidelines weas evaluated using AGREE II. This evaluation included the examination of the guidelines' foundational information alongside key elements of diagnosis, treatment, prevention, and Recommended Grade. The process was conducted by four independent researchers (ZC Wang, MY Lu, R Shi, and J Wang), who assigned scores to each guideline based on the guideline's full text content. These scores, ranging from 1 to 7, reflect the comprehensiveness and quality of the report. Discrepancies in score differences exceeding two points were resolved through group discussion. Finally, scores for each domain were calculated according to the AGREE II reporting checklist's formula, leading to a comprehensive evaluation achieved through consultation.

## Results

### Selection of guidelines

**Figure 1:**
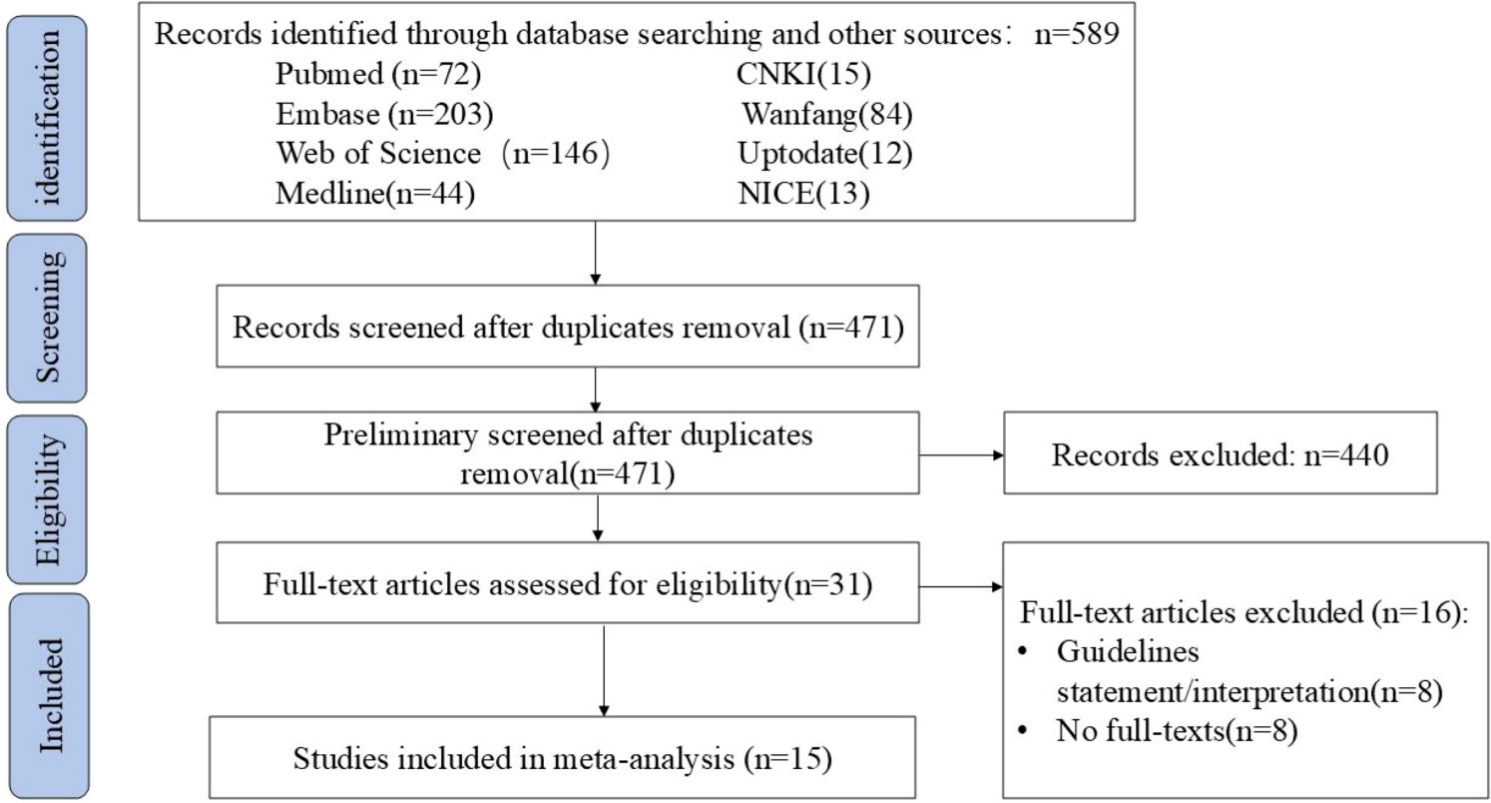
Screening chart of this study.

A total of 15 guidance documents with specific recommendations were eligible, including six consensus statements (13–18) and nine guidelines (1, 19–26). A detailed flow chart of the search and selection is presented in Figure 1.

Two documents were developed in Europe (13, 26), one (14) in Italy, two (15, 16) in China, one (17) in France, four (1, 19, 20, 25) in the United States, one (21) in Germany, one (22) in Japan, one (23) in Canada, one (24) in England, and one (18) is a multinational expert consensus. The guidelines from Inflammatory Bowel Diseases (IOIBD) and the European Society of Coloproctology (ESCP), the Italian society of colorectal surgery (SICCR), the Clinical Practice Guidelines Committee of the American Society of Colon and Rectal Surgeons (ASCRS), German S3, the Japan Society of Coloproctology (JSCP), the Canadian Association of Gastroenterology (CAG), Crohn's disease anal fistula consensus expert group Beijing Anorectal Society, the French National Society of Coloproctology (SNFCP), American Gastroenterological Association (AGA) and Association of Coloproctology of Great Britain and Ireland (ACPGBI). Eight documents (13, 15, 17–20, 23, 25) address perianal fistulizing Crohn's disease (pfCD) population, while three documents (16, 24, 26) focus on anal fistulas population, and four documents (1, 14, 21, 22) on both anal fistulas and abscesses. Screening and diagnostic recommendations are present in twelve documents (1, 13–17, 19, 21–24, 26), with all documents providing guidance on treatment and management. The detailed characteristics of the eligible guidelines and consensus statements are presented in Table 1.

**Table 1:** Guidelines and consensus statement included in this paper.

No.	Guideline	Institution/Group	Language	Year	Target	Screening and diagnosis	Treatment and management	Number of references	Evidence based	Grading of evidence
1	Krisztina B Gecse et al.	Inflammatory Bowel Diseases (IOIBD) and the European Society of Coloproctology (ESCP)	English	2014	Perianal fistulising Crohn's disease	Yes	Yes	127	Yes	Yes
2	David A. Schwartz et al.	Crohn's & Colitis Foundation of America, Inc.	English	2015	Perianal fistulising Crohn's disease	Yes	Yes	35	N/A	No
3	Ficher and Zoccali	Crohn's & Colitis Foundation of America, Inc.	English	2015	Perianal fistulising Crohn's disease	No	Yes	35	Yes	Yes
4	Amato et al.	The Italian society of colorectal surgery (SICCR)	English	2015	Abscess and Anal fistula	Yes	Yes	132	Yes	Yes
5	Andreas Ommer et al.	German S3	English	2017	Abscess and Anal fistula	Yes	Yes	76	Yes	Yes
6	Tetsuo Yamana	The Japan Society of Coloproctology (JSCP)	English	2018	Abscess and Anal fistula	Yes	Yes	65	Yes	No
7	G. Williams et al.	Association of Coloproctology of Great Britain and Ireland (ACPGBI)	English	2018	Anal fistula	Yes	Yes	224	Yes	Yes
8	A. Hillary Steinhart et al.	The Canadian Association of Gastroenterology (CAG)	English	2019	Perianal fistulising Crohn's disease	Yes	Yes	82	Yes	No
9	Lu et al.	Crohn's disease anal fistula consensus expert group	Chinese	2019	Perianal fistulising Crohn's disease	Yes	Yes	71	N/A	Yes
10	Chen et al.	Beijing Anorectal Society	Chinese	2020	Anal fistula	Yes	Yes	22	N/A	Yes
11	D. Bouchard et al.	The French National Society of Coloproctology (SNFCP)	English	2021	Perianal fistulising Crohn's disease	Yes	Yes	16	N/A	No
12	Joseph D. Feuerstein et al.	American Gastroenterological Association (AGA)	English	2021	Perianal fistulising Crohn's disease	No	Yes	26	Yes	Yes
13	Jeroen Geldof et al.	N/A	English	2022	Perianal fistulising Crohn's disease	No	Yes	45	Yes	Yes
14	Wolfgang B et al.	The Clinical Practice Guidelines Committee of the American Society of Colon and Rectal Surgeons (ASCRS)	English	2022	Abscess and Anal fistula	Yes	Yes	267	Yes	Yes
15	Lillian Reza et al.	European Society of Coloproctology (ESCP)	English	2023	Anal fistula	Yes	Yes	148	Yes	No

### Quality assessment

The scores for each domain of the AGREE II instrument are presented in Table 2. The guideline developed by the European Society of Coloproctology (ESCP) achieved the highest score across all six domains (26). Ten guidance documents (1, 13, 17, 18, 21–26) had high scores in the “scope and purpose” domain (domain 1), whereas five documents (14, 19–22) achieved sufficient scores in this domain (average, 65%; range, 45%–80%). The scores for the “rigor of development” (domain 2) were generally low, with only three documents (17, 20, 24) achieving sufficient scores. seven guidance documents (1, 13, 18, 21, 23, 25, 26) had high scores, while five (1, 14, 18, 19, 22) received low scores (domain 2: average, 44%; range, 0%–75%). Eight documents (1, 13, 17, 18, 20, 23, 25, 26) demonstrated high quality in the “stakeholder involvement” domain, three (14, 21, 24) sufficient quality, and the remainder low quality (domain 3: average, 59%; range, 30%–85%). The “clarity of presentation” domain (domain 4) registered the highest scores among the domains (domain 4: average, 65%; range, 38%–88%). Overall, the “applicability” domain (domain 5) had the lowest scores (domain 5: average, 29%; range, 12%–50%). Seven documents (1, 18, 19, 23–26) rated as having sufficient quality in domain 5, whereas others were rated as with low quality. Eleven (1, 13, 14, 17, 18, 20–22, 24–26) were of high quality in the “editorial independence” domain (domain 6: average 51%; range, 0%–88%), one (19) achieved sufficient quality, however, two documents (1, 18) received a score of 0 because they made no mention of conflicts of interest at all.

**Table 2:** Assessment of guidelines and consensus statements by AGREE II.

Guideline	AGREEII score					
	Domain 1 scope and purpose (%)	Domain 2 rigor of development (%)	Domain 3 stakeholder involvement (%)	Domain 4 clarity of presentation (%)	Domain 5 applicability (%)	Domain 6 editorial independence (%)
Krisztina B Gecse et al.	72	68	68	81	32	76
David A. Schwartz et al.	55	30	36	48	38	47
Ficher and Zoccali	57	56	68	82	12	65
Amato et al.	62	33	62	86	17	80
Andreas Ommer et al.	72	66	60	82	40	70
Tetsuo Yamana	68	25	54	60	20	72
A. Hillary Steinhart et al.	75	78	82	78	48	75
Williams Gl et al.	77	56	80	88	27	72
Lu et al.	65	30	42	46	22	0
Chen et al.	64	25	30	40	14	0
D. Bouchard et al.	71	58	58	65	25	78
Joseph D. Feuerstein et al.	77	75	68	64	42	82
Jeroen Geldof et al.	76	68	75	70	38	80
Wolfgang B et al.	78	71	82	84	36	85
Lillian Reza et al.	80	75	85	82	50	88

### Screening and diagnosis

The comprehensive evaluation of the perianal region is crucial not merely for making informed medical and surgical treatment decision-making processes but also significant prognostic value, as evidenced by numerous studies. Twelve guidelines and consensus statements address the screening and diagnosis of anal fistula (1, 13–17, 19, 21–24, 26).

MRI is widely recognized as the preferred diagnostic modality for anal fistula. In the comprehensive articles concentrating on screening and diagnosis, MRI was highly recommended as the preferred diagnostic modality for anal fistula. The evidence supporting this recommendation was of a notably high caliber, achieving a level 1a classification. A specific document (26) emphasizes that examination under anaesthesia (EUA) should not serve as the sole diagnostic tool in complex cases, citing the superiority of MRI and endoanal ultrasound (EAUS). Anorectal endoscopic ultrasound is acknowledged as a viable alternative to MRI in certain guidelines and consensus statements, with two guidelines (1, 26) proposed that transanal ultrasound may serve as an adjunct diagnostic means, providing an additional perspective for accurate evaluation. Five documents (13, 17, 19, 23, 26) reference the potential role of CT imaging in diagnostic workups but explicitly highlight its limitations, including reliance on contrast agents and radiation exposure. Current evidence deems CT insufficiently reliable in this specific application, with formal recommendations advising against its routine use. One guideline (24) notes that CT imaging could serve as a provisional alternative diagnostic approach in scenarios where MRI is unavailable, contingent upon case-specific clinical considerations and resource availability. The Parks anatomical classification remains widely utilized for delineating fistulous tract-sphincter relationships, while the 2019 Chinese consensus (15) endorses the AGA classification system, whereas Japanese guidelines (22) employ the domestically developed Sumikoshi classification.

Furthermore, in documents outlining diagnostic recommendations for pfCD an imposing, four guidelines (13, 15, 16, 19) stated that MRI, anorectal ultrasound, and examination under anaesthesia (EUA) to increase the diagnostic accuracy and optimize treatment strategy. Five guidelines or consensus statements (14, 15, 19, 21, 23) recognize the critical importance of anesthesia-assisted examination for the precise diagnosis and classification of anal fistula. Among all the guidelines, only one guideline (17) concerning Crohn's anal fistula includes endoscopy as part of the diagnostic protocol. A recent expert consensus (18) proposes a disease-specific classification framework for Crohn's disease-associated anal fistulas, emphasizing patient-centered healing objectives, symptom control, and anatomical reparability, contrasted with conventional morphology-driven systems.

### Treatment and management

All guidance documents encompass recommendations for the therapeutic procedures. Treatment and management recommendations are divided into four categories: preoperative management, surgical method selection, drug selection, and postoperative management. Cryptoglandular-origin fistulas and pfCD necessitate distinct clinical approaches due to divergent pathophysiological mechanisms, highlighting the importance of tailored therapeutic algorithms for each entity.

Surgery is the primary treatment modality for perianal fistulas. Fistulotomy is the preferred surgical procedure for patients with simple fistulas. Eight documents refer to fistula excision, with one (1) identifying the risk factors for anal sphincter dysfunction post-excision, such as preoperative fecal incontinence, recurrent fistulas, female gender, complex fistulas, and previous anal rectal surgery. Women with anterior fistulas or prior childbirth-related sphincter damage also face increased risks, leading to recommendations for alternative interventions to preserve sphincter function. And it is proposed that patients with these risk factors adopt other intervention measures apart from fistula excision to preserve function. A total of eight documents are related to placement of the noncutting/loose setons, with one (14) advising against their use due to potential prolonged healing durations and increased post-operative pain. Some guidelines (13, 20) assert the importance of adequate drainage to prevent pelvic sepsis, and it is of most significance to ensure adequate drainage. The use of drainage tubes is advocated to maintain fistula patency, with loose tubes helping to conserve the integrity of the anal sphincter and promote healing. Other guidelines contend that for patients averse to additional surgery, a loose suture can be considered a long-term solution; however, the evidence levels for these recommendations remain low. Three guidelines (1, 21, 24) do not recommend suture ligation as a primary treatment for anal fistula due to the existence of postoperative incontinence. One guideline (16) contends that the cure rate of anal fistula ligation surpasses that of excision, noting that the technique requires further standardization, improvement, and popularization. A total of seven studies (1, 13, 14, 16, 20, 21, 26) have referred to the rectal advancement flap. Specifically, two guidelines (13, 21) explicitly recommend that this surgical technique for pfCD should be administered only after achieving inflammation control. In guidelines published prior to 2020, the evidence grade for this approach consistently remained at Level 1B, whereas the recommendation grades in the 2022 (1) and 2023 (26) guidelines were downgraded to Level 1C. Owing to the paucity of evidence on long-term efficacy and the elevated recurrence rate, three guidelines (1, 21, 26) indicate that fibrin glue constitutes an ineffective treatment for anal fistulas, and the guideline working group consents that fibrin glue should merely be utilized in exceptional circumstances. The recommended attitudes towards the LIFT procedure in guidelines from various countries exhibit significant differences, and the evaluations are mostly based on the surgical success rate and the effectiveness of functional protection. Seven guidelines endorse the LIFT procedure, with one (21) strongly recommend the LIFT procedure as the first-line option for high anal fistulas based on moderate-quality evidence, emphasizing its advantages in terms of success rate and sphincter protection. Although the Chinese consensus (16) acknowledges the short-term efficacy of the LIFT procedure, it points out its limitations in terms of recurrence risk and for patients who are obese or have undergone multiple surgeries. The ASCRS guidelines (1) list it as the preferred surgical procedure for transsphincteric fistulas, but clearly prohibit its use for anal pfCD. In contrast, the ESCP guidelines (26) hold a conservative attitude towards the LIFT procedure, considering that there is insufficient evidence regarding its long-term efficacy. In terms of perioperative management, one guideline (21) stipulates that no special intestinal preparation is required before surgery if the fistula is removed or a drainage tube is placed. It is currently unknown whether pre-surgical bowel cleaning or delaying post-surgical defecation enhances the healing rate. One guideline (16) exists that elaborates on the dietary requirements, post-operative outcomes, and preventive measures for patients post-anorectal fistula surgery, while other guidelines or consensus statements fail to address this aspect. The summary of recommendations for different diagnostic and surgical approaches is presented in Table 3.

**Table 3:** Summary of recommendations for diagnosis and treatment.

Recommendations	Krisztina B Gecse et al. (13)	Amato et al. (14)	Lu et al. (15)	Chen et al. (16)	D. Bouchard et al. (17)	Jeroen Geldof et al. (18)	David A. Schwartz et al. (19)
MRI	+	+	N/A	+	N/A	N/A	+
EUS	+	N/A	N/A	N/A	N/A	N/A	N/A
EUA	+	+	N/A	N/A	N/A	N/A	N/A
CT	+	N/A	N/A	N/A	N/A	N/A	N/A
PDAI Score	+	N/A	N/A	+	N/A	N/A	N/A
Park Classification	+	+	+	N/A	+	N/A	+
Sumikoshi Classification	N/A	N/A		N/A	N/A	N/A	N/A
Loose Setons	+	+	N/A	+	N/A	N/A	N/A
Fistulotomy	+	+	N/A	+	N/A	N/A	N/A
MAF	+	+	N/A	+	N/A	N/A	+
Cutting Setons	N/A	N/A	N/A	N/A	N/A	N/A	N/A
LIFT	N/A	+	N/A	+	N/A	N/A	N/A
Stem Cell Injection	+	N/A	N/A	N/A	N/A	N/A	N/A
Rectal Resection	N/A	N/A	N/A	+	N/A	N/A	N/A
Fibrin Glue	N/A	+	N/A	N/A	N/A	N/A	N/A
Anal Fistula Plug	+	+	N/A	N/A	N/A	N/A	N/A

Recommendations	Ficher and Zoccali (20)	Andreas Ommer et al. (21)	Tetsuo Yamana (22)	A. Hillary Steinhart et al. (23)	G. Williams et al. (24)	Joseph D. Feuerstein et al. (25)	Wolfgang B et al. (1)	Lillian Reza et al. (26)
MRI	N/A	+	N/A	+	+	N/A	+	+
EUS	N/A	N/A	+	N/A	N/A	N/A	N/A	N/A
EUA	+	N/A	N/A	+	N/A	N/A	N/A	N/A
CT	N/A	N/A	+	N/A	+	N/A	−	N/A
PDAI Score	N/A	N/A	N/A	N/A	+	N/A	N/A	N/A
Park Classification	+	+	N/A	N/A	+	N/A	+	N/A
Sumikoshi Classification	N/A	N/A	+	N/A	N/A	N/A	N/A	N/A
Loose Setons	+	+	+	N/A	+	N/A	+	+
Fistulotomy	+	+	N/A	N/A	+	N/A	+	+
MAF	+	+	N/A	N/A	N/A	N/A	+	+
Cutting Setons	N/A	−	+	N/A	+	N/A	+	−
LIFT	+	+	N/A	N/A	+	N/A	+	+
Stem Cell Injection	+	N/A	N/A	N/A	N/A	N/A	N/A	N/A
Rectal Resection	+	N/A	N/A	N/A	N/A	N/A	+	N/A
Fibrin Glue	N/A	−	N/A	N/A	−	N/A	−	−
Anal Fistula Plug	N/A	−	N/A	N/A	−	N/A	−	+

For special types of anal fistulas, relevant management recommendations have been addressed in some guidelines. Extrasphincteric fistulas typically fall into the category of complex or high-level fistulas, which implies the necessity of more cautious surgical strategies. The guidelines (13, 26) emphasize that for extrasphincteric fistulas, infection control should be given priority. It is advisable to consider performing seton drainage to avoid irreversible sphincter damage caused by direct incision, and sphincter-preserving techniques are recommended. A consensus (18) classifies extrasphincteric fistulas as either “Class 2b (symptom control)” or “Class 3 (severe perineal destruction)”, and joint decision-making by gastroenterologists, surgeons, and radiologists is required. If the fistula tract is associated with active rectal inflammation, the inflammation should first be controlled with TNF agents before attempting repair surgery. Rectovaginal fistula (RVF), recognized as a specific subtype of anal fistula, is addressed in partial guidelines. Although its incidence remains low, RVF significantly impairs patients' quality of life. The rectal advancement flap is recommended as the first-line surgical approach by six relevant (1, 14, 20, 21, 24, 26) guidelines. Four guidelines (1, 20, 24, 26) advocate the use of gracilis muscle transplantation for recurrent cases, while two guidelines (1, 24) suggest a trial of 3–6 months of conservative therapy for obstetric RVF.

Guidelines related to pfCD pay more attention to the coordination between drug therapy and surgery. All eight guidelines about pfCD commented on proctectomy and fecal diversion stated that this may be necessary in patients with severe perianal fistulizing disease, but this should be considered as a last resort. Current guidelines and consensus statements uniformly emphasize anti-TNF-α agents as the cornerstone of medical management for Crohn's perianal fistulas. Over half of these guidelines strongly recommend infliximab as first-line therapy based on high-quality evidence at level 1A, demonstrating superior efficacy compared to alternative pharmacological options. Adalimumab is recognized as moderately effective with level 1B evidence. The AGA guidelines (25) endorse short-term antibiotic combination for infection control while explicitly discouraging monotherapy with aminosalicylates or corticosteroids. Regional variations emerge in therapeutic strategies, particularly regarding immunomodulator co-administration. The Chinese consensus (15) advocates mandatory combination therapy of anti-TNF-α agents with immunomodulators to mitigate antidrug antibody formation in Asian populations, contrasting with the French consensus (17) that maintains a neutral stance on adalimumab-immunomodulator combination, supported by only 50% of panel experts. For refractory cases, one consensus proposes tacrolimus as a secondary option, while one documents (13) assert the role of stem cell injection in the treatment of Crohn's disease fistula.

## Discussion

### Key findings

This review provides a comprehensive analysis of the currently available national and international guidelines on the management of perianal fistula, identifying areas of consensus and divergence in recommendations. This comprehensive analysis of 15 international guidelines highlights both evolving consensus and persistent controversies, reflecting geographic, methodological, and evidence-based disparities. Anal fistula is a disease that is notoriously difficult to treat autonomously. This condition poses significant challenges, as it demands a scrupulous and all-encompassing approach. It is of the highest significance to precisely diagnose, efficaciously manage, and meticulously select a fitting treatment plan for this specific disorder. Sufficient attention must be dedicated to every aspect related to it. In the diagnosis and treatment of anal fistulas, guidelines across nations exhibit regional characteristics and discrepancies in evidentiary basis. However, the core consensus encompasses the following: magnetic resonance imaging (MRI) is established as the preferred imaging modality; the combination of anti-tumor necrosis factor (anti-TNF) agents and surgical intervention constitutes the standard protocol for Crohn's disease-associated anal fistulas; and sphincter-preserving techniques are prioritized in clinical practice.

The surgical treatment discussed in the guidelines mainly consists of: fistulotomy, cutting setons, loose setons, advancement flap, fibrin glue/fistula plug, adipose-derived stem cells, and proctectomy. Notably, this study discovered diverse perspectives on setons therapy through the comparison of multiple guidelines. Its application strategies show significant regional differences in different guidelines, reflecting the diversity of evidence quality, medical resources, and clinical traditions. Setons can be classified into cutting setons and non-cutting/loose setons. The issue of incontinence constitutes a crucial concern and an undesirable complication ensuing from cutting setons. Nevertheless, no randomized controlled trials (RCTs) have juxtaposed cutting setons with other repair treatments. The results concerning sphincter incontinence in these studies are debated due to patient heterogeneity in fistula anatomy, sphincter involvement, and surgical history, which might give rise to disparities in reported outcomes and impede the synthesis of results. We assert that the efficacy of cutting setons still demands further exploration. Only one (16) document referred to the criteria for assessing the anus function after an anal fistula operation. Physical examination, questionnaires, ultrasound, and other means can be employed to evaluate the anus function, and the patient's subjective feelings and quality of life are the paramount criteria for assessing the anus function after an anal fistula operation. Despite the evidence being somewhat weak, the recommendation for this evaluative approach remains strong.

A multitude of guidelines consistently address the management of fistulas in Crohn's disease. For patients presenting with signs or symptoms of active fistulizing Crohn's disease, imaging studies (EUS or MRI) are recommended to delineate the anatomical structure of the fistula. In the case where there is evidence of complex fistulizing disease, referral to a surgeon is recommended. In the treatment of fistulizing disease, antibiotics are initially recommended to achieve a symptomatic response, followed by anti-TNF therapy to induce symptomatic response; patients who achieve symptom relief are suggested to continue treatment to achieve and maintain complete remission. All the guidelines regarding the treatment of Crohn's fistula, anti-TNF medications are emphasized as the cornerstone, with infliximab being the most extensively supported (13, 17–20, 23, 25). it should be emphasized that when the pharmacotherapy strategy proves inadequate for symptom response, referral for surgical treatment is recommended.

At present, two versions (1, 27) of guidelines issued by ASCRS exist. The current study exclusively incorporated the 2022 edition of guidelines in its evaluation; however, we assert that a comparative analysis between sequential guideline versions is critical to delineate temporal advancements and discrepancies in clinical recommendations. Such a comparison would further elucidate the evolution of evidence thresholds, therapeutic hierarchies, and regional consensus variations over time, thereby enhancing the interpretability of current practice standards. The 2022 edition revises and augments the 2016 guidelines. The literature encompassed within the 2016 version extends until December 2015, while the literature covered by the 2022 version reaches until November 2021. Both versions utilize a structured GRADE system to appraise the strength of their recommendations, presented through a systematic framework of recommendation and evidence grading. With respect to minimally invasive treatment methods, the 2022 update integrates assessment of minimally invasive techniques, including endoscopic and laser closure methods. In the context of Crohn's disease-related fistulas, the 2022 version underscores the significance of integrating surgical with pharmacological treatments and introduces the application of mesenchymal stem cells. Simultaneously, it documents shifts in the recommendation level for several treatments, noting a downgrade for cutting setons (from 2B to 2C), and upgrades for biological patch and fibrin glue (from 2B to 1B), as well as for LIFT surgery in Crohn's disease (from 2B to 1B).

In 2016, E.J. de Groof et al. (28) pioneered the first systematic analysis of global guidelines on anal fistula. As a groundbreaking study in this field, it comprehensively compared the consensus and controversies across guidelines, established the central role of MRI in anal fistula diagnosis, highlighted the foundational use of anti-TNF agents in Crohn's disease-related anal fistulas, and identified key issues such as the lack of unified standards in the Parks and AGA classification systems. The study's emphasis on “heterogeneous evidence quality” and “needs for multidisciplinary management” laid the foundation for subsequent research. Building upon the 2016 version, this current study expanded the search scope to 2024, incorporating 15 guidelines with particular inclusion of Asian regional guidelines. Notably, the consensus first proposed in 2016—“MRI as the preferred imaging modality”—was further reinforced. New evidence revealed that the combination of MRI and endoscopic ultrasound (EAUS) achieved a 92% diagnostic accuracy for Crohn's disease-related anal fistulas, compared to 85% with MRI alone in 2016, highlighting the value of multimodal imaging. Additionally, the ESCP 2023 guideline included in this study for the first time listed “stem cell injection” as a second-line treatment for complex anal fistulas, whereas the 2016 analysis only mentioned this technique being in the clinical trial phase. This update incorporates the latest evidence on novel surgical techniques and pharmaceutical applications, reflecting advancements in treatment technologies and clinical evidence.

Compared with the single—dimensional evaluation using the Oxford Evidence Level (1a–5) in 2016, this study introduced the AGREE II tool to conduct a quantitative analysis from six dimensions such as “scope and purpose” and “development rigor”. Meanwhile, by applying the AGREE II instrument to evaluate guideline quality, this study found widespread inadequacies in “applicability” and “declaration of conflicts of interest” across guidelines, further revealing real-world barriers to guideline implementation. In this study, we systematically collated anal fistula guidelines and incorporated them into our review. The assessment of the guidelines using the AGREE II tool revealed that the European Society of Coloproctology (ESCP) possess the highest methodological quality of all the included guidelines (26), offering 42 recommendations across seven sections related to the diagnostic and therapeutic management of perianal abscess and cryptoglandular anal fistula. The guideline development process is compliant with the AGREE II tool kit.

“Applicability” constitutes a key indicator for the practical implementation of guidelines and is often limited by the absence of descriptions of enablers and barriers, and a scarcity of additional tools and resources, all of which may exert an influence on health improvement. Future guidelines for anal fistula management merit enhance methodological rigor by strictly adhering to AGREE II criteria, with a focus on developing practical tools such as clinical decision algorithms and fostering multidisciplinary collaboration among stakeholders. High-quality randomized controlled trials incorporating long-term outcome assessments, including recurrence rates, incontinence risk, and quality of life metrics, are essential to address evidence gaps in emerging therapies. Tailored recommendations should be systematically integrated for special populations, such as immunocompromised individuals, pediatric patients, and obstetric cases with sphincter injuries. To ensure clinical relevance and evidence-based practice, guidelines should undergo dynamic revisions every three to five years, supported by real-world data from registry studies.

### Strengths and limitations

The strength of this study lies in its exhaustive and systematic literature review, which was conducted to identify the relevant guidelines and statements for the diagnosis and treatment of anal fistula. Two reviewers independently evaluated these documents using the AGREE II framework, identifying methodological weaknesses, and a lack of implementation strategies and resources, which could inform the development of future guidelines. These recommendations were summarized and compared for consistency and discrepancies among them. The limitations of this study are that, despite the implementation of a systematic search strategy, certain guidelines and consensus statements might have been overlooked due to language barriers. Additionally, the absence of a well-established threshold for scoring AGREE II domain, and reliance on previous publications for domain evaluation, may introduce bias. Although this study increased sample size and optimized evaluation tools, it shares similar limitations with the 2016 research. Future efforts should leverage global multicenter RCTs and interdisciplinary collaboration to further enhance the scientific rigor and clinical applicability of guidelines.

## Conclusions

In conclusion, the existing clinical practice guidelines comprehensively cover the diagnosis and treatment of anal fistulas, with some providing graded recommendations based on the quality of evidence. It is also essential to note the limitations of the current evidence and to continuously gather and analyze clinical experiences to support the ongoing refinement and update of the guidelines. All potentially effective diagnostic and therapeutic methods, encompassing traditional, complementary, and alternative methods, should be meticulously considered. These methods need to be thoroughly evaluated, and solid and persuasive evidence should be furnished to support them. Methodological evaluation via the AGREE II tool revealed significant heterogeneity in guideline rigor, with domains such as “applicability” and “stakeholder involvement” scoring suboptimally. While high-quality guidelines provide comprehensive, evidence-based frameworks, many lack implementation strategies or fail to address critical populations. During the entire process of guideline development, tools such as AGREE II and the Healthcare Practice Guideline Reporting Project (HPGRP) tools can be referred to. These tools provide valuable frameworks and benchmarks to ensure systematic, evidence-based guideline development.

## Data Availability

The original contributions presented in the study are included in the article/Supplementary Material, further inquiries can be directed to the corresponding author.
